# Determining the safety and efficacy of dietary supplementation with 3ˊ-sialyllactose or 6ˊ-sialyllactose on growth, tolerance, and brain sialic acid concentrations

**DOI:** 10.3389/fnut.2023.1278804

**Published:** 2023-10-19

**Authors:** Rebecca K. Golden, Loretta T. Sutkus, Laura L. Bauer, Sharon M. Donovan, Ryan N. Dilger

**Affiliations:** ^1^Neuroscience Program, University of Illinois, Urbana, IL, United States; ^2^Department of Animal Sciences, University of Illinois, Urbana, IL, United States; ^3^Department of Food Science and Human Nutrition, University of Illinois, Urbana, IL, United States; ^4^Division of Nutritional Sciences, University of Illinois, Urbana, IL, United States

**Keywords:** growth, hematology, histomorphology, sialic acid, 3ˊ-sialyllactose, 6ˊ-sialyllactose

## Abstract

Sialylated oligosaccharides, including 3ˊ-sialyllactose (3ˊ-SL) and 6ˊ-sialyllactose (6ˊ-SL), comprise a large portion of human milk and have been known to support development over the first year of life. While research has investigated the impact of early-life supplementation, longer-term supplementation remains relatively unexplored. Consequently, the following study assesses the impact of supplementation of either 3ˊ-SL or 6ˊ-SL on growth performance, tolerance, and brain sialic acid concentrations. Two-day-old piglets (*n* = 75) were randomly assigned to a commercial milk replacer *ad libitum* without or with 3ˊ-SL or 6ˊ-SL (added at 0.2673% on an as-is basis). Daily body weight and feed disappearance were recorded to assess growth performance and tolerance. Pigs were euthanized for sample collection on postnatal day 33 (*n* = 30) or 61 (*n* = 33), respectively. Across growth performance, clinical chemistry and hematology, histomorphology, and sialic acid quantification, dietary differences were largely unremarkable at either time-point. Overall, SA was well-tolerated both short-term and long-term.

## 1. Introduction

Human milk is a complex combination of essential nutrients and bioactive components that are beneficial to the health and cognitive development of the infant. Human milk serves as a source of nutrition and immunologic protection for the first year of life (1, 2). Key components of human milk are human milk oligosaccharides (HMO), which are carbohydrates composed of five main monosaccharides: glucose, galactose, fucose, *N*-acetylglucosamine, and sialic acid (SA) (3). Functioning as prebiotics, antiadhesive antimicrobials, immune modulators, and nutrients for brain development, HMO provide a wide array of beneficial effects for the developing infant (4, 5). Bovine milk contains low concentrations of structurally similar, predominately sialylated oligosaccharides. Thus, infant formula made from bovine milk are typically devoid of oligosaccharides (6). Due to documented benefits of HMO, preclinical and clinical research have investigated supplementation of HMO to formula (7) in hopes of replicating the complex nature of human milk, although isolating the individual benefits of HMO has proven difficult.

Investigating SA-containing compounds has been of interest due to their presence across animal species as well as their prevalence throughout body tissues (8). Specifically, SA are naturally and most frequently found in the fundamental forms of *N*-acetylneuraminic (Neu5Ac) and *N*-glycolylneuraminic (Neu5Gc) acids throughout the body (9, 10). These natural forms of SA are synthesized in various tissues and organs, but it is believed that due to the immature development of the infant at birth, human milk is the primary source during infancy (11, 12). Human milk-derived SAs are found most commonly as components of oligosaccharide chains, with about 69%–76% as gangliosides and 21%–28% as glycoproteins, while only 3% are found in the free form (10). Despite this, infant formulas contain less than 25% of the SA concentration found in human milk, with infant formula compositions varying widely between commercial products (10). Therefore, it is of increasing importance to find suitable ways of supplementing SA into infant formula, whether it be through natural sources or by chemical synthesis (5).

Furthermore, multiple studies have indicated that the majority of ganglioside-bound sialic acids have been found in the brain, as opposed to organs such as the spleen, thyroid, liver, pancreas, heart, and small intestine (8, 13). Hence, much of the literature focuses on how bound SAs may impact brain development as well as overall development of the infant. Recent evidence suggests there are multiple benefits associated with consumption of sialyllactose, specifically in its predominant forms: 3ˊ-sialyllactose (3ˊ-SL) and 6ˊ-sialyllactose (6ˊ-SL) (14). The abundance of individual HMO fluctuates throughout lactation. Whereas 6ˊ-SL is more abundant during the first few months of lactation and decreases over time, the opposite is true for 3ˊ-SL, which increases across lactation (2). These sialylated oligosaccharides have been tested in a wide array of model species (15–20). Specifically, enzymatically synthesized 3ˊ-SL and 6ˊ-SL have been previously shown to be well-tolerated and safe for neonatal pigs in a study that established appropriate dosages for infant formula (16, 17). Tarr et al. (14) also observed that 3ˊ-SL and 6ˊ-SL supplementation in mice was associated with anxiolytic properties, maintenance of mucosa-associated microbiota communities, and prevention of stress-induced neuron reduction. In general, sialylated oligosaccharides are believed to function as “decoys’ to prevent the binding of pathogens, such as rotavirus (21) and cholera toxin, to host cells and hence function as a preventative mechanism against infection in the infant (8). This has been showcased across several studies, where sialyllactose supplementation was shown to decrease signaling and circulating levels of inflammatory cytokines in disease conditions (20, 22).

Though a multitude of studies have explored how supplementation of 3ˊ-SL and 6ˊ-SL affect development, there remains conflicting evidence regarding how SA concentrations in the brain are impacted. Several studies indicate changes of SA concentration in the corpus callosum, cerebellum, prefrontal cortex, and hippocampus associated with supplementation (23, 24). Conversely, other studies indicate that no differences in concentrations were observed in pigs supplemented with sialyllactose (25). Importantly, forms and dosages of sialyllactose supplementation are inconsistent, leading to poor correlation of results across studies. To our knowledge, only short-term supplementation of 3ˊ-SL and 6ˊ-SL has been assessed causing the effects of longer-term supplementation to be unknown.

Therefore, the objective of this study was to evaluate the effects of short-and long-term supplementation of enzymatically synthesized 3ˊ-SL and 6ˊ-SL on growth performance, health outcomes, organ weights, and blood and regional brain SA concentrations in the pig. We hypothesized that both 3ˊ-SL and 6ˊ-SL would be well-tolerated and support normal long-term growth and development in the pig. Based on previous studies, we also predicted that SA concentrations would be increased in sialyllactose supplemented groups at both 4-week and 8-week time-points (23, 24).

## 2. Materials and methods

### 2.1. Animal care and housing

All animal care and experimental procedures listed were approved by the University of Illinois Urbana-Champaign Institutional Animal Care and Use Committee and in accordance with the National Research Council Guide for the Care and Use of Laboratory Animals. A total of 75 intact, non-castrated male pigs were sourced from a commercial swine herd on postnatal day (PND) 2 after receiving colostrum from the sow. The number of pigs utilized in this study provided 80% power to detect 1.25 standard deviation units between treatment groups using a 5% level of significance (i.e., alpha = 0.05). Pig genetics included Pig Improvement Company (PIC; Hendersonville, TN) Line 3 sows that were artificially inseminated using a pooled semen source from 50–150 boars in order to minimize genetic variability. Upon arrival at the University of Illinois Piglet Nutrition and Cognition Laboratory (PNCL), pigs received a 5-mL subcutaneous and a 3-mL oral dose of *Clostridium perfringens* antitoxin C and D (Colorado Serum Company, Denver, CO). The pigs were artificially reared at PNCL until PND 33, at which time 6 pigs (2 per treatment, per cohort) were euthanized and used for baseline sample collection. Longitudinal pigs (3 per treatment, per cohort) were transferred to the Veterinary Medicine Research Farm (VMRF) on PND 33 and raised in group pens until PND 61 [postnatal week (PNW) 8], at which time all remaining pigs were euthanized for sample collection. The study consisted of 5 cohorts each with 15 pigs. Only pigs with a starting body weight of at least 1.4 kg were utilized for this study, as previous studies conducted in PNCL have shown that lower body weight upon arrival increases the probability of failure-to-thrive. Pigs were randomly assigned to one of three treatment conditions in a blinded fashion as described below (Section 2.2). Treatment assignment was randomized considering initial body weight and litter of origin (i.e., genetics) to eliminate bias as a result of these factors.

Custom pig rearing units (individual cage dimensions: 87.6 cm × 88.9 cm × 50.8 cm; L × W × H), described previously in Fil et al. (26), were used to house pigs individually while at PNCL. Cages consisted of vinyl-coated metal flooring, one stainless-steel wall, and three acrylic walls, which allowed pigs to see, hear, and smell each other without directly touching. Environmental conditions at PNCL included a 12 h light/dark lighting schedule, with the ambient, micro-environmental temperature maintained between 26.6–29.0°C throughout the study. Twice-daily health checks were performed to monitor pigs for diarrhea, lethargy, body composition, and any other clinical indicators of failure to thrive. For the first week at PNCL, all pigs, regardless of treatment group, were provided access to electrolytes (Swine BlueLite; TechMix, Stewart, MN or Bounce Back, MannaPro®, LLC, St. Louis, MO) due to incidence of loose stool.

At PND 33, pigs continuing to the final study time-point were transferred to VMRF where they were group-housed by treatment in nursery pens (1.219 m × 1.219 m; vinyl-coated, expanded metal flooring; 1 nipple drinker; *ad libitum* access to feed via trough feeder) until PND 61 when they were humanely euthanized for tissue collection. Once-daily health checks were performed by trained VMRF staff to monitor for signs of injury or failure to grow. All study personnel in direct contact with animals were blinded to treatment groups for the duration of the study and data analyses.

### 2.2. Experimental treatments and feeding paradigm

Pigs were randomly assigned to one of three treatment groups: control (**CON**), control +3ˊ-sialyllactose (**3ˊ-SL**), or control +6ˊ-sialyllactose (**6ˊ-SL**). All supplementation was included at a rate of 0.2673% of the diet on an as-is basis (PND 2–33: 500 mg/L; PND 33–61: 2.673 g/kg). Diets for PND 2–33 were manufactured in powder form by TestDiet (Purina Mills, St. Louis, MO). All diets were based on a commercial milk replacer product (ProNurse^®^ Specialty Milk Replacer, Land O’Lakes, North Arden Hills, MN, United States), and supplemented with either lactose (CON) or the respective sialyllactose test article (3ˊ-SL or 6ˊ-SL) (Table 1). Diets for PND 33–61 were manufactured as practical, nutritionally-adequate, and age-appropriate mash diets, and pigs were maintained within the same experimental dietary group (i.e., CON, 3ˊ-SL, or 6ˊ-SL) (Table 2). Test articles were added to a basal mash formula (corn-soybean meal-based) by a single, unblinded researcher who was not involved in daily handling of pigs. All daily feedings at PNCL and trough fillings at VMRF were done by blinded study personnel.

**Table 1:** Analyzed composition of experimental milk replacer treatments fed through 4 week of age (dry matter and liquid bases)^a^.

		Dietary treatment					
		CON	3ˊ-SL	6ˊ-SL	CON	3ˊ-SL	6ˊ-SL
Dietary form		Powder	Powder	Powder	Liquid	Liquid	Liquid
Item	Units^b^	g/100 g DM	g/100 g DM	g/100 g DM	g/L	g/L	g/L
Dry matter		91.39	91.76	91.71	182.78	183.52	183.42
Organic matter		90.47	90.37	90.35	165.36	165.85	165.72
Ash		9.53	9.63	9.65	17.42	17.67	17.70
Acid hydrolyzed fat		27.94	28.38	28.14	51.07	52.08	51.61
Crude protein		26.49	26.55	26.40	48.42	48.72	48.42
Total AA		25.25	25.60	25.81	46.15	46.98	47.34
Amino acids							
Alanine		1.24	1.24	1.23	2.27	2.28	2.26
Arginine		0.66	0.69	0.69	1.21	1.27	1.27
Aspartic acid		2.56	2.62	2.60	4.68	4.81	4.77
Cysteine		0.56	0.57	0.58	1.02	1.05	1.06
Glutamic acid		4.28	4.29	4.26	7.82	7.87	7.81
Glycine		0.51	0.52	0.51	0.93	0.95	0.94
Histidine		0.49	0.50	0.50	0.90	0.92	0.92
Isoleucine		1.63	1.61	1.59	2.98	2.95	2.92
Leucine		2.59	2.62	2.58	4.72	4.81	4.71
Lysine		2.40	2.30	2.68	4.39	4.22	4.92
Methionine		0.46	0.47	0.47	0.84	0.86	0.86
Phenylalanine		0.83	0.84	0.83	1.52	1.54	1.52
Proline		1.46	1.51	1.50	2.67	2.77	2.75
Serine		1.07	1.12	1.13	1.96	2.06	2.07
Threonine		1.60	1.66	1.64	2.92	3.05	3.01
Tryptophan		0.44	0.46	0.43	0.80	0.84	0.79
Tyrosine		0.53	0.61	0.61	0.97	1.12	1.12
Valine		1.54	1.55	1.54	2.81	2.84	2.82
Neu5Ac^c^, μg/g							
Free		227.5	176.8	194.6	0.042	0.032	0.036
Bound		5,589	6,784	6,823	1.022	1.245	1.251
Total		5,816	6,960	7,018	1.063	1.277	1.287
Neu5Gc^c^, μg/g							
Free		116.6	85.37	86.87	0.021	0.016	0.016
Bound		52.57	75.89	122.5	0.010	0.014	0.022
Total		169.2	161.3	209.3	0.031	0.030	0.038

a Dietary treatment powders were manufactured in powder form by TestDiet (Purina Mills, St. Louis, MO). The composition was identical to ProNurse (Land O’Lakes commercial sow-milk replacer formula), with the exception that the control diet was balanced using lactose. AA, amino acids; CON, control diet; Neu5Ac, N-acetylneuraminic acid; Neu5Gc, N-glycolylneuraminic acid; 3ˊ-SL, 3ˊ-sialyllactose diet; 6ˊ-SL, 6ˊ-sialyllactose diet.

b Units listed in this row do not apply to Neu5Ac or Neu5Gc, which are displayed accordingly.

c Bound sialic acid concentrations calculated as the difference between total and free concentrations.

**Table 2:** Analyzed composition of phased dietary treatments fed from 4-to-8-week of age (dry matter basis)^a^.

	Phase 1			Phase 2			Phase 3		
Item	CON	3ˊ-SL	6ˊ-SL	CON	3ˊ-SL	6ˊ-SL	CON	3ˊ-SL	6ˊ-SL
Dry matter, %	88.60	88.65	88.48	88.33	87.14	88.28	86.71	86.66	86.70
Organic matter, %	93.18	93.08	94.27	92.57	93.53	92.74	93.34	94.15	93.94
Ash, %	6.82	6.92	5.73	7.43	6.47	7.26	6.66	5.85	6.06
Acid hydrolyzed fat, %	6.88	6.81	7.04	7.29	7.44	7.33	7.62	7.68	7.51
Crude protein, %	23.15	21.98	22.41	24.39	24.19	23.15	21.89	23.54	23.49
Total AA, %	22.09	21.51	21.89	23.59	23.23	22.68	22.37	22.78	22.45
Amino acids, %									
Alanine	1.10	1.08	1.11	1.13	1.12	1.11	1.10	1.11	1.13
Arginine	1.24	1.21	2.24	1.50	1.45	1.40	1.41	1.47	1.41
Aspartic acid	2.21	2.14	2.17	2.47	2.40	2.35	2.29	2.33	2.28
Cysteine	0.43	0.41	0.43	0.39	0.38	0.35	0.36	0.38	0.34
Glutamic acid	3.75	3.64	3.73	4.20	4.11	4.02	3.99	4.07	4.00
Glycine	0.83	0.83	0.85	0.93	0.94	0.92	0.91	0.92	0.93
Histidine	0.56	0.55	0.57	0.61	0.59	0.58	0.60	0.60	0.59
Isoleucine	0.98	0.96	0.97	1.11	1.08	1.07	1.04	1.05	1.04
Leucine	1.96	1.92	1.93	1.99	1.95	1.93	1.90	1.94	1.92
Lysine	1.71	1.65	1.64	1.69	1.59	1.60	1.67	1.64	1.65
Methionine	0.45	0.45	0.45	0.47	0.48	0.45	0.47	0.52	0.48
Phenylalanine	1.04	1.00	1.04	1.13	1.12	1.08	1.10	1.12	1.10
Proline	1.25	1.22	1.25	1.28	1.24	1.25	1.25	1.26	1.24
Serine	0.97	0.95	1.01	1.04	0.99	1.00	0.89	0.94	0.88
Threonine	1.06	1.03	0.99	0.99	1.19	0.99	0.96	0.95	0.95
Tryptophan	0.29	0.30	0.29	0.28	0.34	0.30	0.26	0.26	0.28
Tyrosine	0.72	0.70	0.71	0.77	0.74	0.74	0.70	0.73	0.69
Valine	1.19	1.16	1.16	1.20	1.18	1.16	1.15	1.16	1.16
Neu5Ac^b^, μg/g									
Free	154.7	197.5	131.0	101.9	104.2	143.1	60.60	97.28	124.3
Bound	1,237	2,555	2,137	747.0	1,978	1,938	1,075	1,586	1,732
Total	1,392	2,753	2,268	848.9	2,082	2,081	1,136	1,684	1,856
Neu5Gc^b^, μg/g									
Free	14.65	14.33	10.74	8.30	5.95	7.04	12.27	4.28	5.10
Bound	250.4	213.3	175.1	8.20	8.54	3.02	0.60	7.60	13.43
Total	265.1	227.7	185.8	16.50	14.49	10.06	12.87	11.88	18.53

a Phase 1 diet was fed for the first week after pigs were transferred to the Veterinary Medicine Research Farm (VMRF). Phase 2 diet was fed the second week at VMRF while Phase 3 was fed until study conclusion. Test articles were incorporated into solid diets manufactured at the University of Illinois. AA, amino acids; CON, control diet; Neu5Ac, N-acetylneuraminic acid; Neu5Gc, N-glycolylneuraminic acid; 3ˊ-SL, 3ˊ-sialyllactose diet; 6ˊ-SL, 6ˊ-sialyllactose diet.

b Bound sialic acid concentrations calculated as the difference between total and free concentrations.

For PND 2–33, liquid milk replacer was reconstituted fresh daily at 200 g of dry powder per 800 mL of tap water. Pigs were on an *ad libitum,* 20-h feeding schedule from 1,000 h to 0600 h the following day. Milk reservoirs were cleaned daily before refilling with freshly reconstituted milk replacer. Milk disappearance was calculated per individual pig based on the initial and final reservoir weights. Milk replacer allotments per pig were based on milk disappearance from the preceding day, ensuring a sufficient amount to allow for *ad libitum* feeding with minimal wastage. More milk was added during the second daily health check, if necessary, with the initial weight (i.e., before adding more milk) and added weight being recorded and considered as part of the daily milk disappearance calculation.

For PND 33–61, pigs received age-appropriate, practical mash diets that were fed for 7, 7, and 14 d for phases 1, 2, and 3, respectively; these represented the standard 3-phase series of dietary formulations used by the University of Illinois Swine Research Center. These diets were composed (inclusion range denoted per successive phase) predominantly of corn (42%, 42%, and 59%), soybean meal (22%, 32%, and 30%), and whey (25%, 20%, and 5%) and were designed to transition pigs from liquid to mash feed. Mash feed was distributed via troughs that allowed multiple pigs to eat at the same time and feed intake was calculated on a pen basis during the PND 33–61 study phase.

### 2.3. Growth measures and health checks

A total of 30 pigs (9 CON; 11 3ˊ-SL; 10 6ˊ-SL) completed the study at the 4-week time-point and 33 pigs (11 CON; 12 3ˊ-SL; 10 6ˊ-SL) continued to the 8-week time-point (i.e., study conclusion). Pigs were excluded in cases of failure to thrive. As part of daily health checks at PNCL, body weights (BW) were recorded once-daily from PND 2–33. Trained study personnel also observed and recorded fecal consistency per pig twice-daily while pigs were at PNCL. Fecal consistency was graded on a scale of 1 (solid, pellet-like) to 4 (liquid diarrhea). Once transferred to VMRF, BW was captured once weekly for the remainder of the study (PNW 4–8). Fecal scoring was not recorded at VMRF as the pigs were group-housed.

Average daily gain (ADG), average daily feed intake (ADFI), and average gain-to-feed ratio (G:F; measure of feed efficiency) were all calculated using the milk disappearance records described in section 2.2 and BW recorded as described above. All measures were calculated on a weekly basis, as well as overall basis, starting on PND 5 at PNCL. Measures were also calculated on weekly and overall bases at VMRF. Overall measures were calculated per housing location (i.e., PNCL and VMRF separately) as separated by the early (PNW 4) and final (PNW 8) study time-points.

### 2.4. Tissue and blood collection

Tissue collection days (PND 33 and PND 61) began at 0900 h. Pigs were anesthetized through an intramuscular injection of a telazol:ketamine:xylazine solution [50.0 mg tiletamine plus 50.0 mg of zolazepam reconstituted with 2.50 mL ketamine (100 g/L) and 2.50 mL xylazine (100 g/L); Fort Dodge Animal Health, Overland Park, KS], with the compounded solution administered at 0.06 mL/kg of BW. To ensure moderate levels of anesthesia, eye and hind hoof reflexes were checked. Once properly sedated, intracardiac blood draws were performed. Blood for plasma extraction was collected into 2 mL evacuated tubes (K2 EDTA, Becton, Dickinson and Company, Franklin Lakes, NJ) and immediately placed on ice. Blood for serum extraction was collected into a 2 mL evacuated tube (Becton, Dickinson and Company, Franklin Lakes, NJ) and left at room temperature for 30 min before being placed on ice. The serum tube and one plasma tube were submitted to the Veterinary Diagnostic Laboratory (College of Veterinary Medicine, University of Illinois at Urbana-Champaign, Urbana, IL) for clinical chemistry and hematology analyses, respectively. Additional blood tubes for plasma extraction were centrifuged at 4°C and 2,250–2,300 × g for 15 min (Allegra 6R centrifuge, Beckman Coulter Life Sciences, Indianapolis, IN), with the resulting plasma aliquoted into cryovials and frozen at −80°C; these plasma samples were designated for SA quantification. After blood collection, pigs were euthanized through an intracardiac injection of sodium pentobarbital (Euthasol, Virbac, Westlake, TX) at 0.22 mL/kg of BW. Immediately following euthanasia, the brain and liver were extracted and weighed before dissection. Additionally, the intestinal tract was removed and segments were dissected to permit samplings from the ileum and ascending colon.

After weighing, a small piece of the right lobe of the liver was minced and flash-frozen in liquid nitrogen. Four regions of interest (ROI) were bilaterally dissected from the brain (cerebellum, hippocampus, prefrontal cortex, and striatum) following a validated procedure (27). Both halves of the ROI were minced together to homogenize the sample. The tissue was then distributed equally among multiple cryovials and flash-frozen in liquid nitrogen. Brain tissue, liver tissue, and plasma samples were stored at −80°C pending further processing. One 5–10 cm tissue section of both the ileum and ascending colon were collected, placed in 10% neutral buffered formalin (NBF), and left at room temperature for less than 24 h before being stored at 4°C until histomorphological analysis. Histomorphological tissue processing and staining was performed by a board-certified veterinary pathologist (Veterinary Diagnostic Pathology, LLC., Fort Valley, VA) who remained blinded to treatment identity at all times.

### 2.5. Sialic acid quantification

Free, total, and bound SA concentrations were quantified in all dietary treatments (both milk replacer and mash feeds per feeding phase), four brain regions (cerebellum, hippocampus, prefrontal cortex, and striatum), and plasma. Standards for Neu5Ac and Neu5Gc were run prior to the study samples. Blinded study personnel analyzed all samples in duplicate.

For diet and brain samples, approximately 0.3 g of each sample was placed into 2 mL locking cap microcentrifuge tubes along with 0.9 mL of phosphate buffered saline (PBS; 1:3 ratio of tissue to PBS) and a stainless steel bead. Samples were homogenized utilizing a bead homogenization system (TissueLyser, Qiagen, Hilgden, Germany) for a total of 1 min before the bead was removed and homogenized samples were stored at −20°C until further analysis.

#### 2.5.1. Free sialic acid preparation

Excluding plasma samples, all free SA samples were prepared by aliquoting 0.2 mL of the homogenized sample and diluting with 0.8 mL of double-distilled water before being vortexed. After vortexing, 0.5 mL of the homogenate or plasma was aliquoted into a 2 mL microfuge tube with 0.5 mL acetonitrile before being vortexed again. After freezing overnight, the samples were then centrifuged for 15 min at 16,500 × g to deproteinize the extracts. From there, aliquots of the liquid phase were transferred to centrifugal filter units (10 kDA molecular weight cut-off) and centrifuged in a swinging bucket rotor for approximately 30 min at 4,000 × g to generate purified extracts.

#### 2.5.2. Total sialic acid preparation

Total SA samples were prepared by aliquoting 0.1 mL (diet and brain samples) or 0.25 mL (plasma samples) of the sample and diluting with 0.9 mL or 1 mL (diet/brain or plasma, respectively) of double-distilled water before being vortexed. Once vortexed, 0.2 mL of the homogenate was combined with 0.3 mL sodium acetate:enzyme solution 1 [1 U neuraminidase +20 mU/mL sodium acetate buffer (4.1 g sodium acetate anhydrous dissolved in water +0.5 mL glacial acetic acid + distilled water, balanced to a pH of 5.0 with glacial acetic acid)] for diet and plasma samples or 0.3 mL sodium acetate:enzyme solution 2 (1 U neuraminidase +40 mU/mL sodium acetate buffer) for brain tissue samples. Samples were then incubated at 37°C for 18 h in a water bath, and then combined with 0.5 mL of acetonitrile, vortexed, and frozen overnight. Once thawed, samples were transferred to centrifugal filter units (10 kDA molecular weight cut-off), and centrifuged in a swinging bucket rotor for approximately 30 min at 4,000 × g to generate purified extracts.

#### 2.5.3. Free, Total, and bound sialic acid quantification

Following sample preparation, free and total concentrations of Neu5Ac and Neu5Gc acids were obtained by ion chromatography with pulsed amperometric detection (ICS-5000, Dionex, Sunnyvale, CA) with a gold cell electrochemical detector and a multi-step gradient of 7–300 mM sodium acetate in 100 mM sodium hydroxide at 30°C. Separation was conducted utilizing a CarboPac PA20 Column, 3 × 150 mm (Dionex, Sunnyvale, CA) with a flow rate of 0.5 mL/min and a 10 μL injection volume (28, 29). Free and total sialic acid for both diet and tissue samples were determined by multiplying the sample hydrolyzed value by the appropriate dilution factor and as-is sample weight and dividing this value by the sample weight (as-is for tissue samples, dry matter-basis for diet samples). For plasma samples, the sample hydrolyzed value was multiplied by the appropriate dilution factor and divided by the sample volume. Concentrations of bound SA were determined by subtracting free SA from the total SA concentrations for each subject.

### 2.6. Statistical analysis

All outcomes were analyzed in SAS 9.3 (RRID:SCR_008567; SAS Inst. Inc., Cary, NC) using a one-way analysis of variance (ANOVA). Depending on the outcome, one of two statistical models was used: (1) any data collected at a single time-point was analyzed with a simple ANOVA using the MIXED procedure to assess the main effect of dietary treatment, or (2) any within-subject data collected at multiple time-points (e.g., daily and weekly BW) was analyzed with a repeated-measures ANOVA using the MIXED procedure. The individual pig served as the experimental unit for all outcomes other than growth performance from week 4–8, where pen served as the experimental unit. Outliers were determined via Studentized residuals, with any data point generating a Studentized residual with an absolute value of 3 or greater being removed. For all statistical methods, main effects were assessed between the three dietary treatments and cohort was included as a blocking factor with litter nested within cohort. The level of significance was set at *p <* 0.05 for all statistical comparisons.

## 3. Results

### 3.1. Health outcomes

#### 3.1.1. Pig body weight

**Figure 1:**
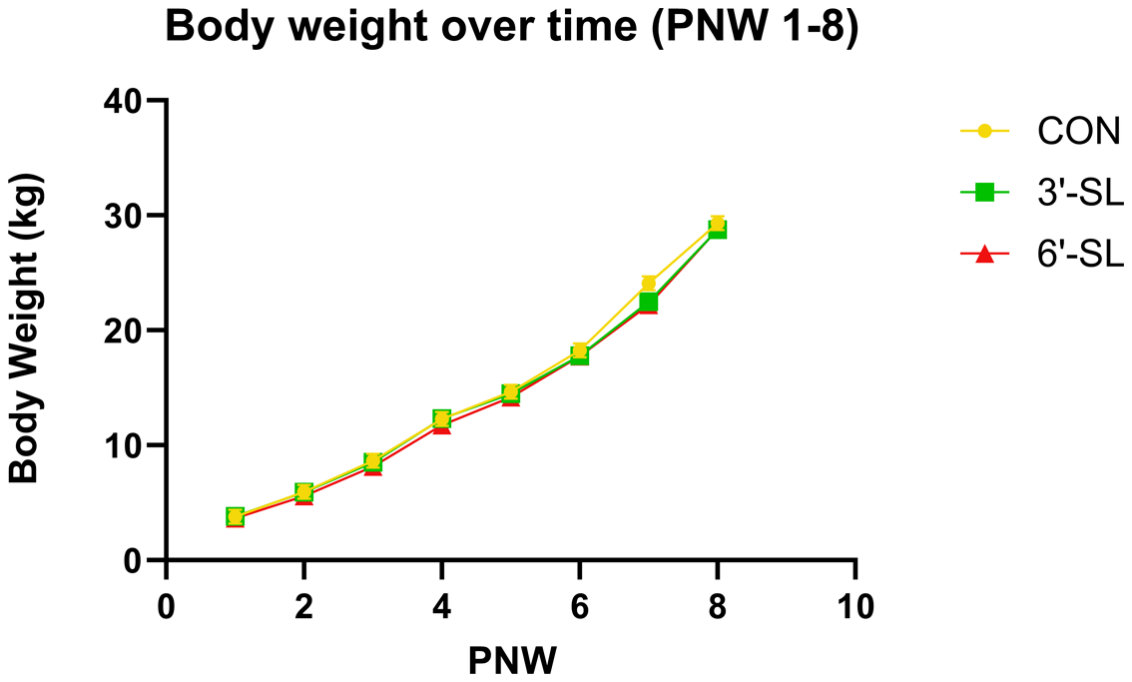
Longitudinal Body Weights. Weekly body weights of pigs on the control (CON), 3ˊ-sialyllactose (3ˊ-SL), and 6ˊ-sialyllactose (6ˊ-SL) diets for the entire 8-week study (weeks 1–8) Data are expressed as least square means with standard error of mean. PNW, postnatal week.

A main effect for postnatal week (PNW) on body weight was observed (*p <* 0.001) for the entirety of the study duration (Figure 1), confirming that pigs were in an active phase of growth. Body weights from 64 pigs were included in the daily BW analysis from PND 2–33. A main effect of PND was observed for daily body weights (*p <* 0.001) (Supplemental Figure S1), but there was no main effect of diet for daily BW (*p =* 0.311) and no interaction (*p =* 0.975) between diet and PND in the first half of the study. Body weights from 26 pigs were included in the weekly BW analysis for PND 34–61 (PNW 4–8). A main effect for PNW was observed for weekly body weights (*p <* 0.001) (Supplemental Figure S2), but there was no main effect of diet on weekly body weights (*p =* 0.444) and no interaction (*p =* 0.642) between diet and PNW in the second half of the study.

#### 3.1.2. Growth performance

Effects of diet on average daily gain (ADG), average daily feed intake (ADFI) on liquid, powder, and mash bases, and gain-to-feed ratio (G:F) are shown in Table 3. No main effect of diet was observed for any growth outcome, where individual body weights were averaged per pen starting at PND 34. No main effect of diet was observed for any growth performance outcomes.

**Table 3:** Growth performance of pigs^a^.

	Diet			Pooled SEM	
Outcome	CON	3ˊ-SL	6ˊ-SL		*p*-value
PND 2–33^b^					
*n*	20	23	21	–	–
ADG, g/d					
PND 5–12	197.4	202.2	167.4	13.0	0.110
PND 12–19	318.3	304.1	298.8	23.8	0.538
PND 19–26	388.3	371.0	375.7	20.1	0.704
PND 26–33	524.9	498.9	516.3	32.3	0.542
Overall	351.0	345.3	341.5	12.6	0.708
ADFI (liquid), g/d					
PND 5–12	1,004	1,013	944	57.1	0.483
PND 12–19	1,582	1,479	1,456	127.6	0.421
PND 19–26	2,309	2,280	2,221	151.1	0.720
PND 26–33	3,115	3,047	2,947	140.7	0.217
Overall	1,987	1,950	1,881	70.9	0.393
Overall ADFI (powder), g/day	397.4	390.0	376.2	14.2	0.393
G:F, g BW:g liquid					
PND 5–12	0.197	0.198	0.183	0.009	0.334
PND 12–19	0.207	0.206	0.208	0.008	0.987
PND 19–26	0.168	0.169	0.181	0.006	0.226
PND 26–33	0.171	0.166	0.177	0.010	0.393
Overall	0.181	0.178	0.183	0.006	0.426
Overall G:F, g BW:g powder	0.903	0.892	0.914	0.032	0.554
PND 34–61^b^					
*n*	3	3	3	–	–
ADG, g/d					
PND 34–41	345.1	321.4	321.9	46.46	0.895
PND 41–48	504.1	474.8	525.8	52.40	0.798
PND 48–55	826.2	846.2	909.3	71.22	0.704
PND 55–61	674.6	896.8	928.6	84.99	0.185
Overall	587.5	592.1	603.6	22.86	0.534

a Growth performance outcomes were calculated starting on PND 5 as pigs were still acclimating to the environment from PND 2–4. CON, control; 3ˊ-SL, 3ˊ-sialyllactose; 6ˊ-SL, 6ˊ-sialyllactose; ADG, average daily gain; ADFI, average daily feed intake; G:F, gain-to-feed (i.e., feed efficiency); PND, postnatal day; SEM, standard error of the mean.

b For PND 2–33, piglets were housed individually and body weights and feed intake data were captured on an individual basis. 3For PND 34–61, piglets were housed in pens by diets. Body weights were captured individually and averaged within pen while feed intake was captured on a per-pen basis.

#### 3.1.3. Fecal scores

**Figure 2:**
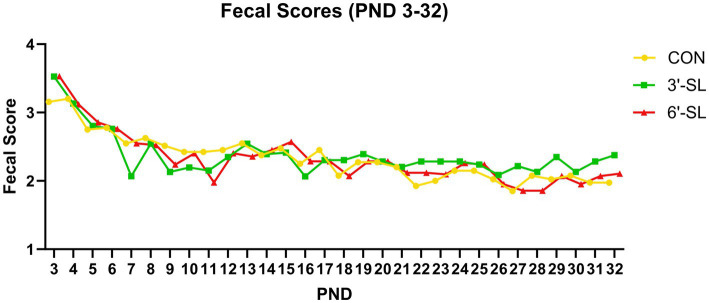
Longitudinal Fecal Scores. Daily fecal scores of pigs on the control (CON), 3ˊ-sialyllactose (3ˊ-SL), and 6ˊ-sialyllactose (6ˊ-SL) diets for the first 4 week. of the study. Fecal scores are expressed as the mean score across time for each treatment. Standard error is not expressed as these were subjective ratings reported on a scale from 1–4. PND, postnatal day.

Fecal consistency data were collected on individual pigs from PND 2–33 as shown in Figure 2. A main effect of PND was observed for daily fecal scores (*p <* 0.001), signifying that fecal consistency became more solid over the first phase of the study where dietary treatments were provided as liquid milk replacer. There was also a main effect of diet for daily fecal scores (*p =* 0.045), with pigs receiving 3ˊ-SL having more solid feces than those assigned to CON and 6ˊ-SL treatments, with no difference between CON and 6ˊ-SL groups. No interaction effect (*p =* 0.915) was observed between diet and PND for daily fecal scores. As stated, starting on PND 34, quantifying fecal scores from individual pigs was no longer possible as pigs were group-housed based on their assigned dietary treatment.

#### 3.1.4. Organ weights

The effects of dietary treatment on whole brain and liver weights at PND 33 and 61 are displayed in Table 4. No main effect of diet was observed on PND 33, but the main effect of diet was observed on PND 61 for absolute brain weight (*p =* 0.035), where 6ˊ-SL pigs had lower (*p <* 0.05) brain weights compared with CON pigs, and 3ˊ-SL pigs were intermediary. However, when expressed relative to body weight, no dietary treatment differences were observed.

**Table 4:** Organ weights at PND 33 and 61^1^.

	Diet			Pooled SEM	
	CON	3ˊ-SL	6ˊ-SL		*p*-value
PND 33					
*n*	9	9	9	–	–
BW, kg	12.07	11.35	11.33	0.587	0.606
Absolute weight, g					
Brain	54.1	51.3	53.9	0.001	0.164
Liver	395.2	378.5	368.5	0.018	0.578
Relative weight, % of BW					
Brain	0.455	0.456	0.488	0.025	0.568
Liver	3.27	3.32	3.26	0.172	0.954
PND 61					
*n*	11	11	10	–	–
BW, kg	28.77	29.05	29.51	1.258	0.913
Absolute weight, g					
Brain	73.2^a^	70.4^ab^	67.4^b^	0.002	0.035
Liver	832.7	892.7	867.0	0.033	0.402
Relative weight, % of BW					
Brain	0.257	0.247	0.234	0.01	0.283
Liver	2.89	3.08	2.97	0.11	0.320

^1^CON, control; 3ˊ-SL, 3ˊ-sialyllactose; 6ˊ-SL, 6ˊ-sialyllactose; BW, body weight; PND, postnatal day; SEM, standard error of mean. ^ab^Means lacking a common superscript letter within a row differ (*p* < 0.05).

### 3.2. Blood and tissue outcomes

#### 3.2.1. Blood chemistry and hematology

The effects of dietary treatment on clinical chemistry and hematology at PND 33 are displayed in Supplemental Table S1. A main effect of dietary treatment (*p <* 0.05) was only observed for total alkaline phosphatase, γ-glutamyl transferase (GGT), and lymphocyte percentage. Known reference intervals for pigs are included, when available, and outcomes where dietary effects were noted remained within expected limits.

The effects of dietary treatment on clinical chemistry and hematology at PND 61 are displayed in Supplemental Table S2. A main effect of dietary treatment was observed for creatine, creatine phosphokinase (CPK), monocyte percentage, and eosinophil percentage. Known reference intervals for pigs are included, where available, for comparison. All outcomes of notable dietary differences remained within the expected limits.

#### 3.2.2. Intestinal histomorphology

The effects of dietary treatment on intestinal structure at PND 33 and PND 61 are displayed in Table 5. No main effects of dietary treatment were observed for any outcomes.

**Table 5:** Intestinal histomorphology at PND 33 and 61^1^.

	Diet			Pooled SEM	
Outcome	Control	3ˊ-SL	6ˊ-SL		*p*-value
PND 33					
*n*	9	11	10	–	–
Ileum					
Villus length, μm	484.8	495.7	500.3	23.77	0.883
Crypt depth, μm	226.5	232.0	231.6	10.25	0.875
Villus width, μm	141.7	145.0	145.8	5.89	0.866
Crypt width, μm	49.55	50.27	47.27	1.52	0.286
V:C ratio	2.25	2.23	2.25	0.12	0.991
Villus surface area, μm^2^	219,386	223,784	231,815	17,792	0.848
Crypt area, μm^2^	10,994	11,598	10,864	601.8	0.615
Ascending colon					
Crypt depth, μm	369.8	392.1	362.8	16.91	0.419
Crypt width, μm	54.39	58.97	57.31	1.73	0.079
Crypt area, μm^2^	20,179	23,186	20,693	1,253.6	0.182
PND 61					
*n*	10	12	10	–	–
Ileum					
Villus length, μm	386.2	406.0	411.7	22.33	0.699
Crypt depth, μm	254.0	252.6	261.8	16.09	0.905
Villus width, μm	144.9	143.3	156.3	8.95	0.487
Crypt width, μm	47.68	46.33	48.18	1.65	0.670
V:C ratio	1.64	1.69	1.68	0.13	0.963
Villus surface area, μm^2^	174,308	181,285	199,414	13,354	0.353
Crypt area, μm^2^	11,176	11,811	12,663	906.14	0.497
Ascending colon					
Crypt depth, μm	385.6	401.9	418.2	14.90	0.320
Crypt width, μm	61.12	62.23	58.17	2.15	0.234
Crypt area, μm^2^	23,585	24,619	24,024	1,093.4	0.760

^1^CON, control; 3ˊ-SL, 3ˊ-sialyllactose; 6ˊ-SL, 6ˊ-sialyllactose; SEM, standard error of mean; V:C, villus length-to-crypt depth ratio.

### 3.3. Sialic acid concentrations

**Figure 3:**
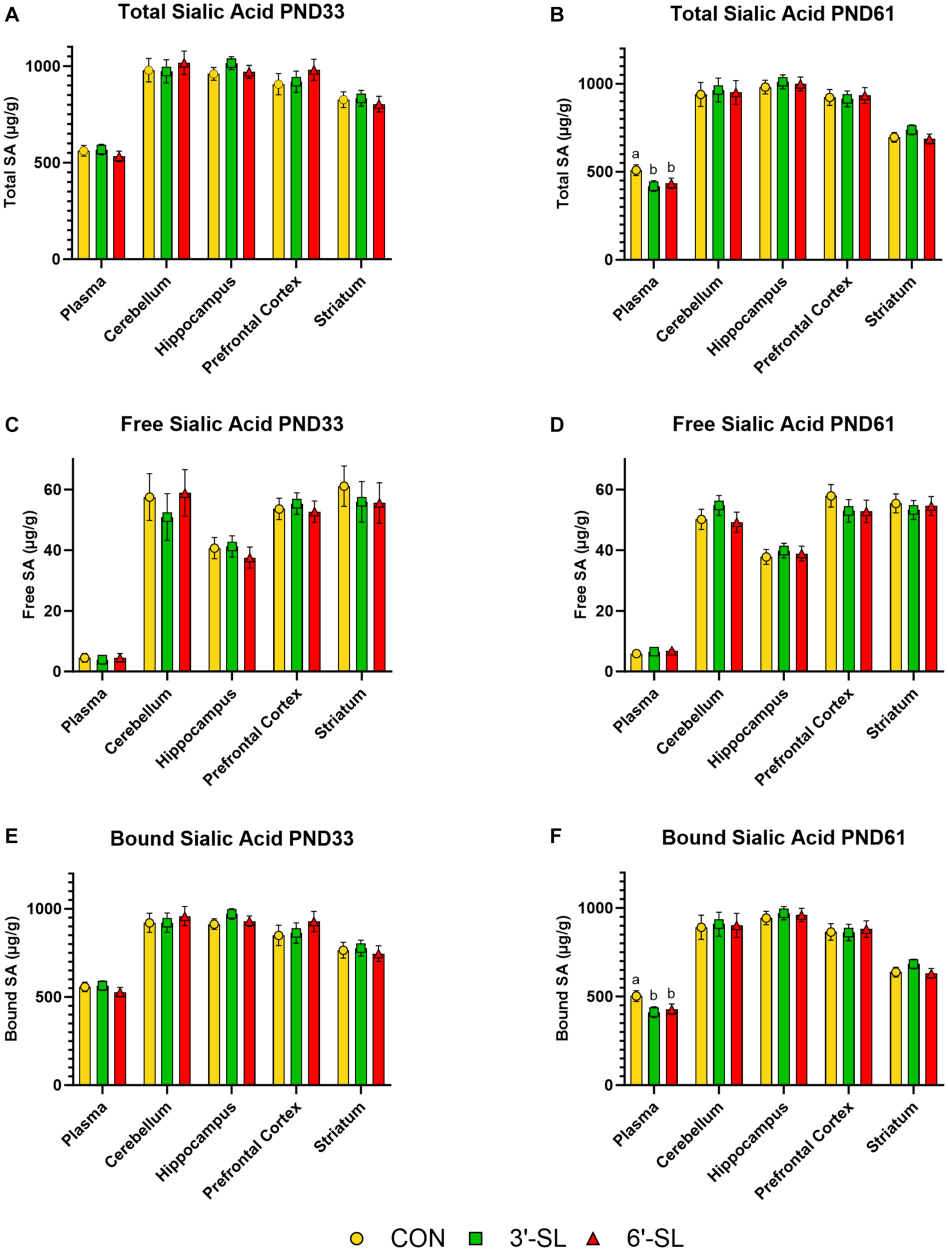
Longitudinal Plasma and Tissue Sialic Acid Concentrations. Total, free, and bound *N*-acetylneuraminic acid (Neu5Ac) concentrations in plasma and brain tissue are displayed for PND 33 **(A,C,E)**. Dietary SL does not influence (*p* > 0.05) total, free, or bound sialic acid concentrations at PND 33. Total, free, and bound Neu5Ac concentrations in plasma and brain tissue are displayed for PND 61 **(B,D,F)**. Dietary SL does influence (*p <* 0.05) total and bound, but not free, sialic acid in plasma, with CON pigs having higher concentrations. Sialic acid supplementation does not influence concentrations in any brain regions at PND 61. Data are expressed as least square means with standard error of mean. PND, postnatal day, SL, sialyllactose.

Free, bound, and total Neu5Ac concentrations in plasma and selected brain regions are displayed separately for PND 33 and PND 61 in Figure 3. Furthermore, the full breakdown of free, bound, and total Neu5Ac and Neu5Gc across plasma and selected brain regions can be found in Supplemental Tables S3, S4. No differences due to dietary treatment were observed for any outcome at the 4-week time-point. A treatment effect was observed for bound and total Neu5Ac acid in plasma (*p =* 0.022) for 8-week-old pigs, where CON pigs had higher (*p <* 0.05) concentrations than either the 3ˊ-SL or 6ˊ-SL groups. No other differences were observed across Neu5Ac and Neu5Gc at the 8-week time-point.

## 4. Discussion

Human milk contains a variety of oligosaccharides, including those containing SA, as fermentable substrates. Human milk contains SA in the form of sialyllactose at an average concentration of 500 mg/L (30), which, along with previous SA supplementation studies (16, 17), was the informing factor for the level used in the current study. However, unlike human milk, typical infant formulas contain between 65–290 mg of sialyllactose/L (31). Testing supplementation at a concentration more similar to that of human milk is imperative to continued efforts of closing the developmental gap between breastfed and formula-fed infants. That said, previous research on SA supplementation has caused contradicting views on the impact that SA-containing sources may have on development. Additionally, the literature is missing direct evidence using biomedical models that replicate the duration of HMO prevalence that occurs across the lactation period. To our knowledge, this is the first study to assess long-term supplementation of 3ˊ-SL or 6ˊ-SL in a translational animal model.

In the present study, diet progression mimicked translational feed stages of human infants. In the first 4 weeks of life, pigs were provided reconstituted (i.e., liquid) milk replacer supplemented with SL sources, similar to how human infants receive either infant formula or human milk from lactating mothers. The experimental milk replacer treatments provided more than twice the concentration of SL compared to the subsequent diet phase, thereby mimicking colostrum and mother’s first milk, which contains the highest concentration of sialylated milk oligosaccharides (4). This trend of higher levels of SL near the beginning of lactation is observed across a wide range of species, including pigs (32). Notably, it has been found that sialylated oligosaccharides dominate overall porcine milk oligosaccharides, whereas for humans, fucosylated structures are the predominant source (33). Despite this, porcine lactation follows a similar pattern to that of human lactation, where the concentration of overall milk oligosaccharides decreases by 36% across lactation (4, 33). In our supplementation study, the inclusion levels of 3ˊ-SL and 6ˊ-SL were kept constant over both feeding phases. As such, pigs were switched from liquid milk replacer beginning on PND 33, when they were started on plant-based (i.e., corn and soybean meal) diets with decreasing concentrations of whey across subsequent weeks. This phased/transitional approach loosely mimics an infant’s transition to solid foods between 6–12 months of age, where the degree of dietary lactose gradually decreases (34).

All pigs, regardless of assigned treatment, grew similarly across each feeding phase and the complete study duration. These results are congruent with previous studies, which also indicated a lack of difference in overall growth performance due to supplementation of 3ˊ-SL or 6ˊ-SL in pigs (16, 17, 23, 35). In terms of body tissues, brain and liver weights for all treatments at PND 33 were also similar to those from previous studies (16, 17, 36). Despite 6ˊ-SL pigs having heavier absolute brain weights at PND 61, brain and liver weights relative to body weight did not differ by dietary treatment, which indicates similarities in allometric organ growth. To our knowledge, there is no previous research suggesting that SL supplementation modifies body or organ growth patterns in pigs, and in fact, it has been found that 3ˊ-SL and 6ˊ-SL supplementation is well-tolerated in pigs (16, 17). Given that sialyllactose is a non-digestible dietary component and that the primary goal of SA is to act as neuronal or microbial support, it is not surprising that supplementation did not affect growth trajectories of young pigs.

Clinical chemistry and hematology results exhibited minor, inconsistent differences between treatment groups at both study time-points. Results were consistent with known reference intervals for specific markers (37). Given that the only difference in diet was the amount and type of SL supplemented, these results are expected as supplementation of SL has not been shown to alter levels of common blood biomarkers (16, 17). The lack of difference in blood chemistry profiles is supportive of SL being a safe supplemental option for infant formula.

Similarly, no differences were observed between treatments for intestinal histomorphological results. While previous literature investigating 3’SL and 6’SL focused on microscopic histological analyses (16, 17), the current study focused on intestinal structure and the capacity for nutrient absorption, as quantified through villus height and crypt depth measurements. The ileum compromises about 4%–5% of the small intestine in young pigs and is an essential site of digestion and absorption for vitamins, minerals, water, and bile acids (38, 39), due to being covered in villi-forming enterocytes, which synthesize enzymes critical for digestion of dietary components. Conversely, the ascending colon is part of the large intestine, which is devoid of digestive and absorptive capacities, but contains colonocytes that organize into crypts. The ascending colon is one of the primary locations of fermentation, where microbial byproducts, such as short-chain fatty acids, are produced through bacterial fermentation and impact epithelial cells (i.e., butyrate), or are subsequently absorbed (acetate, propionate) (38). Crypts vary in size and depth across both the small and large intestine and are said to contribute to maintaining the gastrointestinal barrier integrity (38). Findings reported in our study are congruent with those published by Monaco et al. (40) that assessed different sialyllactose supplementation levels in pigs up to 3 weeks of age. Additionally, previous research completed on 3-to 4-week-old pigs has indicated ileal villi length to average 430–600 μm, crypt depth between 140–270 μm, and villus:crypt ratio between 1.8–2.3 across various conditions, confirming that the values obtained in this study are within expected ranges (41, 42).

Since sialyllactose is a non-digestible component, it passes through the small intestine without being digested or absorbed by the host (43, 44). Specifically, sialyllactoses, such as 3ˊ-SL and 6ˊ-SL, have been found to resist enzymatic and hydrolytic digestions and reach the colon intact, with less than 3% release of free sialic acid from dietary sources (44). Previously, ascending colon crypt depth and area for 3-week pigs were found to range between 240–270 μm and 8–11 mm^2^, respectively, after sialyllactose supplementation (40). Although our results differed slightly, no treatment differences were observed, indicating similar intestinal development and gastrointestinal barrier maintenance in young pigs. Additionally, since it has been determined that less than 3% of free sialic acid is released from 3ˊ-SL and 6ˊ-SL, minimal impact to the colon was expected. In the colon, various bacterial strains are responsible for the breakdown and metabolism of sialic acid to produce byproducts, such as short-chain fatty acids, that can be absorbed by the body (45). From our study, we concluded that 3ˊ-SL and 6ˊ-SL were well-tolerated across time due to a lack of differences across measured histomorphological outcomes between treatment groups at each study time-point. Overall, histological results from our study, which were largely unremarkable, corroborate previous research stating that ingestion of various sources of sialyllactose does not alter intestinal structure at a gross level.

Sialic acid concentrations in various body parts are of interest due to their wide range of beneficial properties, as previously mentioned. Specifically, Neu5Ac and Neu5Gc, commonly referred to as “sialic acids”, are most frequently expressed across various mammalian tissues and organs (10, 46). In the brain, SA is thought of as a building block of gangliosides as well as a key component of neural cell membranes, specifically in regard to neurodevelopment (32). Additionally, SA in the form of sialyllactose stimulates the growth and metabolism of beneficial bacteria (e.g., *Bifidobacterium*), increases intestinal colonization, and benefits maturation of the intestine and immune system (32, 45). Therefore, determining how SA in the forms of Neu5Ac and Neu5Gc are concentrated throughout the body can provide context on potential sites of action.

Previous work by Ji et al. (46) found that younger pigs (PND 3) had higher concentrations of SA in various body tissues and organs, such as the liver, heart, and kidneys, with SA concentrations decreasing with age (up to PND 180). While this study did not quantify SA levels in the brain, our results are consistent with the idea that SA levels decrease with age, as most brain regions from our study contained lower levels at PND 61 than PND 33. Moreover, our findings contradict previous literature in that we did not find differences in SA concentrations in various brain regions between treatments (23, 24). In the case of Jacobi et al., this may be due to differences in supplementation rate. While Jacobi and colleagues supplemented at a rate of 2000 or 4,000 mg SL/L until PND 21, our study supplemented at a rate of 500 mg SL/L. The dosage for the current study was determine by averaging the concentrations of both 3ˊ-SL and 6ˊ-SL in human milk across lactation stages (47). Supplementation of SL has previously been associated with neurological development, memory formation, and cognitive development, and also shown to protect against cell death in the brain (23, 24, 48, 49). As such, providing supplementation during critical growth periods of the brain is crucial. In pigs, that growth spurt occurs at 4 weeks of age (50). While pigs have variable susceptibility to SA, especially in early life, this critical brain growth period was the target of our investigation.

Our plasma SA concentration results contradict previous work that found SA-supplemented pigs had higher Neu5Ac and Neu5Gc levels than their non-supplemented counterparts (51). The scientific literature is divided in interpretation of increased blood SA levels with some suggesting that higher levels are associated with preventing cell death and reducing inflammatory markers (51, 52). Others suggest that increased levels are associated with clinical conditions such as cancers, liver disease, and diabetes (53–55). Previous literature has also alluded to the effects of SA being more apparent with gene expression, specifically in transcriptional changes and metabolic profiles (56). Supplementation with sialylated milk oligosaccharides was observed to be associated with higher lipid-derived metabolite concentrations in the liver and serum of fasted mice, indicating modulated metabolism, as well as altered metabolic profiles in the brain (56). Thus, it is possible that 3ˊ-SL or 6ˊ-SL supplementation may lead to systemic or gene expression changes even if elevated blood SA concentrations were not detected.

Furthermore, the mode of delivery and absorption rates of SA must be considered when interpreting SA concentrations. It has been reported that SA in its free form is more rapidly absorbed than SA in a bound form (e.g., oligosaccharide) (43). Compared to human milk, infant formulas have more bioavailable SA due to smaller milk fat globules (43). This was found to be true across 13 powdered infant formulas. However, in infant formulas, indirect sources of SA, such as whey protein, were utilized, whereas the current study utilized enzymatically derived (i.e., essentially pure) 3ˊ-SL and 6ˊ-SL. Furthermore, it is possible that even though SA may be more bioavailable, it is not reflected in tissue and blood concentrations. Rodent and human studies have suggested that up to 50% of orally-dosed sialyllactose fed to fasted mice is unchanged and excreted via urine within 24 h of dosing with only 1% of the initial dose being detectable in the body at that time, while up to 40% of free Neu5Ac and most Neu5Gc were found to exit the human body via urine within 48 h of oral dosing (57–59). At the same time, researchers conducting a piglet study found that 120 min after an intravenous administration of Neu5Ac, up to 80% of it had dissipated, as determined by regular blood sampling (13). It is possible that the 3-h period of food deprivation that preceded blood collection in our study altered detectable concentrations of SA in blood and tissues. In addition, it is important to consider that effectively pure forms of 3ˊ-SL and 6ˊ-SL were supplemented in our study, rather than providing SA as part of more complex dietary components, which is typically the case with infant formulas.

## 5. Conclusion

Our study showed no negative effects from the addition of SA in the form of 3ˊ-SL or 6ˊ-SL over an 8-week period in young pigs. Pigs grew and developed at similar rates, regardless of source or level of SA supplementation. Additionally, sialic acid concentrations in plasma and brain were not impacted by 3ˊ-SL or 6ˊ-SL supplementation. Overall, long-term supplementation of sialylated milk oligosaccharides was determined to be safe and well-tolerated.

## Data availability statement

The datasets presented in this article are not readily available because commercial products were tested. Requests to access the datasets should be directed to RD, rdilger2@illinois.edu.

## Ethics statement

The animal study was approved by University of Illinois Institutional Animal Care and Use Committee. The study was conducted in accordance with the local legislation and institutional requirements.

## Author contributions

RG: Data curation, Formal analysis, Investigation, Methodology, Visualization, Writing – original draft, Writing – review & editing. LS: Data curation, Formal analysis, Investigation, Methodology, Visualization, Writing – original draft, Writing – review & editing. LB: Methodology, Writing – review & editing. SD: Conceptualization, Funding acquisition, Writing – review & editing. RD: Conceptualization, Data curation, Funding acquisition, Methodology, Project administration, Supervision, Visualization, Writing – review & editing.

## References

[1] Fewtrell MS Morgan JB Duggan C Gunnlaugsson G Hibberd PL Lucas A. Optimal duration of exclusive breastfeeding: what is the evidence to support current recommendations? Am J Clin Nutr. (2007) 85:635S–8S. doi: 10.1093/ajcn/85.2.635s, PMID: 17284769

[2] Plows JF Berger PK Jones RB Alderete TL Yonemitsu C Najera JA. Longitudinal changes in human Milk oligosaccharides (HMOs) over the course of 24 months of lactation. J Nutr. (2021) 151:876–82. doi: 10.1093/jn/nxaa427, PMID: 33693851 PMC8030713

[3] Bode L. Human milk oligosaccharides: every baby needs a sugar mama. Glycobiology. (2012) 22:1147–62. doi: 10.1093/glycob/cws074, PMID: 22513036 PMC3406618

[4] Andreas NJ Kampmann B Mehring Le-Doare K. Human breast milk: a review on its composition and bioactivity. Early Hum Dev. (2015) 91:629–35. doi: 10.1016/j.earlhumdev.2015.08.013 26375355

[5] Bode L. Recent advances on structure, metabolism, and function of human milk oligosaccharides. J Nutr. (2006) 136:2127–30. doi: 10.1093/jn/136.8.2127, PMID: 16857829

[6] Akkerman R Faas MM de Vos P. Non-digestible carbohydrates in infant formula as substitution for human milk oligosaccharide functions: effects on microbiota and gut maturation. Crit Rev Food Sci Nutr. (2019) 59:1486–97. doi: 10.1080/10408398.2017.1414030 29333864

[7] Hill DR Chow JM Buck RH. Multifunctional benefits of prevalent HMOs: implications for infant health. Nutrients. (2021) 13:3364. doi: 10.3390/nu13103364, PMID: 34684364 PMC8539508

[8] Wang B Brand-Miller J. The role and potential of sialic acid in human nutrition. Eur J Clin Nutr. (2003) 57:1351–69. doi: 10.1038/sj.ejcn.1601704 14576748

[9] Schauer R Kelm S Reuter G Roggentin P Shaw L. Biochemistry and role of sialic acids In: Biology of the Sialic Acids. New York: Springer (1995). (pp. 7–49). doi: 10.1007/978-1-4757-9504-2

[10] Wang B Brand-Miller J McVeagh P Petocz P. Concentration and distribution of sialic acid in human milk and infant formulas. Am J Clin Nutr. (2001) 74:510–5. doi: 10.1093/ajcn/74.4.510, PMID: 11566650

[11] Lis-Kuberka J Orczyk-Pawiłowicz M. Sialylated oligosaccharides and glycoconjugates of human milk. The impact on infant and newborn protection, development and well-being. Nutrients. (2019) 11:1–23. doi: 10.3390/nu11020306, PMID: 30717166 PMC6413137

[12] Wang B. Sialic acid is an essential nutrient for brain development and cognition. Annu Rev Nutr. (2009) 29:177–222. doi: 10.1146/annurev.nutr.28.061807.155515, PMID: 19575597

[13] Wang B Downing JA Petocz P Brand-Miller J Bryden WL. Metabolic fate of intravenously administered N-acetylneuraminic acid-6-14C in newborn piglets. Asia Pac J Clin Nutr. (2007) 16:110–5. PMID: 17215187

[14] Tarr AJ Galley JD Fisher S Chichlowski M Berg BM Bailey MT. The prebiotics 3′Sialyllactose and 6′Sialyllactose diminish stressor-induced anxiety-like behavior and colonic microbiota alterations: evidence for effects on the gut-brain axis. Brain Behav Immun. (2015) 50:166–77. doi: 10.1016/j.bbi.2015.06.025.The, PMID: 26144888 PMC4631662

[15] Hauser J Pisa E Arias Vásquez A Tomasi F Traversa A Chiodi V. Sialylated human milk oligosaccharides program cognitive development through a non-genomic transmission mode. Mol Psychiatry. (2021) 26:2854–71. doi: 10.1038/s41380-021-01054-9, PMID: 33664475 PMC8505264

[16] Monaco MH Gurung RB Donovan SM. Safety evaluation of 3′-sialyllactose sodium salt supplementation on growth and clinical parameters in neonatal piglets. Regul Toxicol Pharmacol. (2019) 101:57–64. doi: 10.1016/j.yrtph.2018.11.008 30453008

[17] Monaco MH Kim DH Gurung RB Donovan SM. Evaluation of 6′-sialyllactose sodium salt supplementation to formula on growth and clinical parameters in neonatal piglets. Nutrients. (2020) 12:1030. doi: 10.3390/nu12041030 32283716 PMC7230961

[18] Oliveros E Vázquez E Barranco A Ramírez M Gruart A Delgado-García JM. Sialic acid and sialylated oligosaccharide supplementation during lactation improves learning and memory in rats. Nutrients. (2018) 10:1–16. doi: 10.3390/nu10101519, PMID: 30332832 PMC6212975

[19] Pisa E Martire A Chiodi V Traversa A Caputo V Hauser J. Exposure to 3′sialyllactose-poor milk during lactation impairs cognitive capabilities in adulthood. Nutrients. (2021) 13:4191. doi: 10.3390/nu13124191, PMID: 34959743 PMC8707534

[20] Sodhi C. P. Wipf P. Yamaguchi Y. Fulton W. B. Kovler M. Niño D. F.. (2021). The human milk oligosaccharides 2′-fucosyllactose and 6′-sialyllactose protect against the development of necrotizing enterocolitis by inhibiting toll-like receptor 4 signaling. Pediatr Res. 89:91–101. doi: 10.1038/s41390-020-0852-3 32221473 PMC7529714

[21] Hester SN Chen X Li M Monaco MH Comstock SS Kuhlenschmidt TB. Human milk oligosaccharides inhibit rotavirus infectivity in vitro and in acutely infected piglets. Br J Nutr. (2013) 110:1233–42. doi: 10.1017/S0007114513000391, PMID: 23442265

[22] Duan Q Chen D Yu B Huang Z Luo Y Zheng P. Effect of sialyllactose on growth performance and intestinal epithelium functions in weaned pigs challenged by enterotoxigenic *Escherichia Coli*. Journal of Animal Science and Biotechnology. (2022) 13:30–12. doi: 10.1186/s40104-022-00673-8, PMID: 35236420 PMC8892705

[23] Jacobi SK Yatsunenko T Li D Dasgupta S Yu RK Berg BM. Dietary isomers of sialyllactose increase ganglioside sialic acid concentrations in the corpus callosum and cerebellum and modulate the colonic microbiota of formula-fed piglets. J Nutr. (2016) 146:200–8. doi: 10.3945/jn.115.220152, PMID: 26701794

[24] Mudd AT Fleming SA Labhart B Chichlowski M Berg BM Donovan SM. Dietary sialyllactose influences sialic acid concentrations in the prefrontal cortex and magnetic resonance imaging measures in corpus callosum of young pigs. Nutrients. (2017) 9:1297. doi: 10.3390/nu9121297, PMID: 29182578 PMC5748748

[25] Obelitz-Ryom K Bering SB Overgaard SH Eskildsen SF Ringgaard S Olesen JL. Bovine milk oligosaccharides with sialyllactose improves cognition in preterm pigs. Nutrients. (2019) 11:1–20. doi: 10.3390/nu11061335, PMID: 31207876 PMC6628371

[26] Fil JE Joung S Hayes CA Dilger RN. Influence of rearing environment on longitudinal brain development, object recognition memory, and exploratory behaviors in the domestic pig (*Sus scrofa*). Front Neurosci. (2021) 15:1–16. doi: 10.3389/fnins.2021.649536, PMID: 33841090 PMC8024486

[27] Fleming SA Monaikul S Mudd AT Jacob R Dilger RN. Extraction and Dissection of the Domesticated Pig Brain. J Vis Exp. (2021) 170:e62030. doi: 10.3791/62030 33970130

[28] Rohrer JS. Analysis of sialic acids using high-performance anion-exchange chromatography. Anal Biochem. (2000) 283:3–9. doi: 10.1006/abio.2000.4643 10929801

[29] Thermo Fisher Scientific, (2016). Direct determination of sialic acids in glycoprotein Hydrolyzates by HPAE-PAD. Application Update 180. 1–9. Available at: https://tools.thermofisher.com/content/sfs/brochures/AU-180-IC-Sialic-Acids-Glycoprotein-Hydrolyzates-AU71730-EN.pdf

[30] Martín-Sosa S Martín MJ García-Pardo LA Hueso P. Distribution of sialic acids in the Milk of Spanish mothers of full term infants during lactation. J Pediatr Gastroenterol Nutr. (2004) 39:499–503. doi: 10.1097/00005176-200411000-00010, PMID: 15572889

[31] Spichtig V Michaud J Austin S. Determination of sialic acids in milks and milk-based products. Anal Biochem. (2010) 405:28–40. doi: 10.1016/j.ab.2010.06.010, PMID: 20553868

[32] Jahan M Francis N Wynn P Wang B. The potential for sialic acid and sialylated glycoconjugates as feed additives to enhance pig health and production. Animals. (2021) 11:1–19. doi: 10.3390/ani11082318, PMID: 34438776 PMC8388453

[33] Wei J Wang ZA Wang B Jahan M Wang Z Wynn PC. Characterization of porcine milk oligosaccharides over lactation between primiparous and multiparous female pigs. Sci Rep. (2018) 8:4688–16. doi: 10.1038/s41598-018-23025-x, PMID: 29549280 PMC5856818

[34] Cichero JAY. Introducing solid foods using baby-led weaning vs. spoon-feeding: a focus on oral development, nutrient intake and quality of research to bring balance to the debate. British Nutr Found Nutr Bull. (2016):72–7. doi: 10.1111/nbu.12191

[35] Fleming SA Chichlowski M Berg BM Donovan SM Dilger RN. Dietary sialyllactose does not influence measures of recognition memory or diurnal activity in the young pig. Nutrients. (2018) 10:1–12. doi: 10.3390/nu10040395, PMID: 29570610 PMC5946180

[36] Vu VH Donovan SM Brink LR Li Q Gross G Dilger RN. Developing a reference database for typical body and organ growth of the artificially reared pig as a biomedical research model. Front Pediatr. (2021) 9:1–12. doi: 10.3389/fped.2021.746471, PMID: 34926340 PMC8672453

[37] Ventrella D Dondi F Barone F Serafini F Elmi A Giunti M. The biomedical piglet: establishing reference intervals for haematology and clinical chemistry parameters of two age groups with and without iron supplementation. BMC Vet Res. (2017) 13:1–8. doi: 10.1186/s12917-017-0946-2, PMID: 28095847 PMC5240404

[38] Laerke HN Hedemann MS. The digestive system of the pig In: Nutritional physiology of pigs: Videncenter for Svineproduktion (2012). 1–27.

[39] Wilfart A Montagne L Simmins PH Van Milgen J Noblet J. Sites of nutrient digestion in growing pigs: effect of dietary fiber. J Anim Sci. (2007) 85:976–83. doi: 10.2527/jas.2006-431 17121971

[40] Monaco MH Wang M Pan X Li Q Richards JD Chichlowski M. Evaluation of sialyllactose supplementation of a prebiotic-containing formula on growth, intestinal development, and bacterial colonization in the neonatal piglet. Current Developments in Nutrition. (2018) 2:1–15. doi: 10.1093/cdn/nzy067, PMID: 30443641 PMC6226774

[41] Daniels VC Wang M Hirvonen J Jensen HM Ouwehand AC Mukherjea R. Evaluation of 2’-Fucosyllactose and *Bifidobacterium longum* subspecies infantis on growth, organ weights, and intestinal development of piglets. Nutrients. (2022) 14:1–12. doi: 10.3390/nu1401019 PMC8747721 35011074

[42] Sommer KM Jespersen JC Sutkus LT Lee Y Donovan SM Dilger RN. Oral gamma-cyclodextrin-encapsulated tributyrin supplementation in young pigs with experimentally induced colitis. J Anim Sci. (2022) 100:1–10. doi: 10.1093/jas/skac314, PMID: 36161319 PMC9671115

[43] Claumarchirant L Sanchez-Siles LM Matencio E Alegría A Lagarda MJ. Evaluation of sialic acid in infant feeding: contents and bioavailability. J Agric Food Chem. (2016) 64:8333–42. doi: 10.1021/acs.jafc.6b03273 27750424

[44] Engfer MB Stahl B Finke B Sawatzki G Daniel H. Human milk oligosaccharides are resistant to enzymatic hydrolysis in the upper gastrointestinal tract. Am J Clin Nutr. (2000) 71:1589–96. doi: 10.1093/ajcn/71.6.1589, PMID: 10837303

[45] Coker JK Moyne O Rodionov DA Zengler K. Carbohydrates great and small, from dietary fiber to sialic acids: how glycans influence the gut microbiome and affect human health. Gut Microbes. (2021) 13:1–18. doi: 10.1080/19490976.2020.1869502, PMID: 33615984 PMC7899658

[46] Ji S Wang F Chen Y Yang C Zhang P Zhang X. Developmental changes in the level of free and conjugated sialic acids, Neu5Ac, Neu5Gc and KDN in different organs of pig: a LC-MS/MS quantitative analyses. Glycoconj J. (2017) 34:21–30. doi: 10.1007/s10719-016-9724-9, PMID: 27613535

[47] Soyyilmaz B Mikš MH Röhrig CH Matwiejuk M Meszaros-matwiejuk A Vigsnæs LK. The mean of milk: a review of human milk oligosaccharide concentrations throughout lactation. Nutrients. (2021) 13:2737, MDPI AG. doi: 10.3390/nu13082737, PMID: 34444897 PMC8398195

[48] Ferrari G Batistatou A Greene LA. Gangliosides rescue neuronal cells from death after trophic factor deprivation. J Neurosci. (1993) 13:1879–87. doi: 10.1523/jneurosci.13-05-01879.1993, PMID: 8478681 PMC6576579

[49] Rahmann H. Hirnganglioside und Ged ichtnisbildung. Naturwissenschaften. (1994) 81:7–20. doi: 10.1007/BF01138555 8127376

[50] Conrad MS Dilger RN Johnson RW. Brain growth of the domestic pig (*Sus scrofa*) from 2 to 24 weeks of age: a longitudinal MRI study. Dev Neurosci. (2012) 34:291–8. doi: 10.1159/000339311, PMID: 22777003 PMC3646377

[51] Park DB Kim L Hwang JH Kim K-T Park JE Choi J-S. Temporal quantitative profiling of sialyllactoses and sialic acids after oral administration of sialyllactose to mini-pigs with osteoarthritis. RSC Adv. (2023) 13:1115–24. doi: 10.1039/d2ra05912f, PMID: 36686942 PMC9811936

[52] Goehring KC Marriage BJ Oliver JS Wilder JA Barrett EG Buck RH. Similar to those who are breastfed, infants fed a formula containing 2′-fucosyllactose have lower inflammatory cytokines in a randomized controlled trial. J Nutr. (2016) 146:2559–66. doi: 10.3945/jn.116.236919, PMID: 27798337

[53] Crook MA Tutt P Simpson H Pickup JC. Serum sialic acid and acute phase proteins in type 1 and type 2 diabetetes mellitus. Clin Chim Acta. (1993) 219:131–8. doi: 10.1016/0009-8981(93)90204-H, PMID: 7508342

[54] Hogan-Ryan A Fennelly JJ Jones M Cantwell B Duffy MJ. Serum sialic acid and Cea concentrations in human breast Cancer. Br J Cancer. (1980) 41:587–92. doi: 10.1038/bjc.1980.101, PMID: 7387856 PMC2010272

[55] Stefenelli N Klotz H Engel A Bauer P. Serum sialic acid in malignant tumors, bacterial infections, and chronic liver diseases. J Cancer Res Clin Oncol. (1985) 109:55–9. doi: 10.1007/BF01884255, PMID: 3871778 PMC12253832

[56] Charbonneau MR O’Donnell D Blanton LV Totten SM Davis JCC Barratt MJ. Sialylated Milk oligosaccharides promote microbiota-dependent growth in models of infant undernutrition. Cells. (2016) 164:859–71. doi: 10.1016/j.cell.2016.01.024, PMID: 26898329 PMC4793393

[57] Tangvoranuntakul P Gagneux P Diaz S Bardor M Varki N Varki A. Human uptake and incorporation of an immunogenic nonhuman dietary sialic acid. Proc Natl Acad Sci U S A. (2003) 100:12045–50. doi: 10.1073/pnas.2131556100, PMID: 14523234 PMC218710

[58] ten Bruggencate SJ Bovee-Oudenhoven IM Feitsma AL van Hoffen E Schoterman MH. Functional role and mechanisms of sialyllactose and other sialylated milk oligosaccharides. Nutr Rev. (2014) 72:377–89. doi: 10.1111/nure.12106, PMID: 24828428

[59] Nöhle U Schauer R. Metabolism of sialic acids from exogeneously administered sialyllactose and mucin in mouse and rat. Hoppe-Seyler’s Zeitschrift Fur Physiologische Chemie. (1984) 365:1457–68. doi: 10.1515/bchm2.1984.365.2.1457, PMID: 6526381

