# Supplementary material for: Determining the safety and efficacy of dietary supplementation with 3ˊ-sialyllactose or 6ˊ-sialyllactose on growth, tolerance, and brain sialic acid concentrations
Source: Front Nutr. 2023 Oct 19;10:1278804. doi: 10.3389/fnut.2023.1278804 (PMC10620723; doi:10.3389/fnut.2023.1278804)
Supplement: Supplementary file 1 [file Data_Sheet_1.docx]

**Supplemental Figure 1.** Daily body weights of pigs on the control (CON), 3`-siallylactose (3`-SL), and 6`-siallylactose (6`-SL) diets for the first 4 wk of the study. Abbreviations: PND, postnatal day.

**Supplemental Figure 2.** Weekly body weights of pigs on the control (CON), 3`-siallylactose (3`-SL), and 6`-siallylactose (6`-SL) diets for the last 4 wk of study. Abbreviations: PNW, postnatal week.

| **Supplemental Table 1.** Blood clinical chemistry and hematology outcomes at PND 33^1^ | | | | | |  |
| --- | --- | --- | --- | --- | --- | --- |
|  | **Diet** | | | **Pooled SEM** |  | **Reference Interval^2^** |
| **Outcome** | **CON** | **3`-SL** | **6`-SL** |  | ***P*-value** |  |
| *n*^3^ | 9 | 11 | 10 | - | - | - |
| Clinical chemistry |  |  |  |  |  |  |
| Creatine, mg/mL | 0.65 | 0.62 | 0.62 | 0.03 | 0.569 | 0.51-1.39 |
| BUN, mg/dL | 8.09 | 8.51 | 9.37 | 0.61 | 0.137 | 4.0-39 |
| Total protein, g/dL | 4.32 | 4.32 | 4.25 | 0.15 | 0.680 | 2.5-6.6 |
| Albumin, g/dL | 3.19 | 3.15 | 3.22 | 0.07 | 0.733 | 1.9-4 |
| Globulin, g/dL | 1.19 | 1.20 | 1.06 | 0.10 | 0.317 | - |
| Albumin:globulin ratio | 2.68 | 2.75 | 3.05 | 0.24 | 0.405 | 0.7-2.2 |
| Calcium, mg/dL | 10.48 | 10.25 | 10.57 | 0.14 | 0.196 | 7.1-11.6 |
| Phosphorus, mg/dL | 11.29 | 11.09 | 10.65 | 0.20 | 0.082 | 5.3-9.6 |
| Sodium, mmol/L | 138.8 | 137.8 | 137.8 | 1.14 | 0.360 | 125-147 |
| Potassium, mmol/L | 4.49 | 4.43 | 4.52 | 0.15 | 0.887 | 2.9-4.6 |
| Sodium:potassium ratio | 31.22 | 31.45 | 31.10 | 1.07 | 0.967 | - |
| Chloride, mmol/L | 101.5 | 101.2 | 100.7 | 1.06 | 0.582 | 94-106 |
| Glucose, mg/dL | 138.3 | 147.6 | 148.9 | 5.38 | 0.318 | 34-159 |
| Total alkaline phosphatase, U/L | 387.6^a^ | 282.6^b^ | 367.2^a^ | 34.0 | 0.021 | 110-1292 |
| AST, U/L | 30.33 | 44.91 | 39.50 | 8.42 | 0.448 | 32-84 |
| GGT, U/L | 34.64^ab^ | 28.84^b^ | 39.94^a^ | 4.33 | 0.047 | 10-60 |
| Total bilirubin, mg/dL | 0.17 | 0.15 | 0.15 | 0.03 | 0.461 | 0-1.0 |
| CPK, U/L | 627.6 | 934.3 | 705.3 | 169.7 | 0.238 | 61-1251 |
| Cholesterol total mg/dL | 67.78 | 56.55 | 63.80 | 3.86 | 0.108 | 36-54 |
| GLDH, U/L | 0.71 | 1.00 | 0.82 | 0.22 | 0.167 | - |
| Bicarbonate (TCO_2_), mmol/L | 26.94 | 26.80 | 27.41 | 0.81 | 0.767 | 18-27 |
| Magnesium, mg/dL | 2.28 | 2.21 | 2.15 | 0.08 | 0.071 | - |
| Triglycerides, mg/dL | 17.11 | 13.36 | 14.13 | 2.16 | 0.384 | - |
| Anion gap | 14.87 | 14.33 | 14.32 | 0.69 | 0.698 | - |
| Hematology |  |  |  |  |  |  |
| RBC count, ×10^6^ cells/uL | 5.79 | 5.51 | 5.57 | 0.17 | 0.350 | 4.08-8.17 |
| Hemoglobin, g/dL | 8.51 | 8.13 | 8.35 | 0.27 | 0.580 | 4.32-13.3 |
| Hematocrit, % | 29.27 | 27.88 | 28.31 | 0.78 | 0.427 | 16-41 |
| MCV, fL | 50.68 | 50.73 | 50.97 | 1.11 | 0.948 | 34.2-61.3 |
| MCH, pg | 14.72 | 14.73 | 14.98 | 0.38 | 0.733 | 9.4-19.8 |
| MCHC, g/dL | 28.94 | 29.01 | 29.37 | 0.36 | 0.452 | 26.5-33.6 |
| NRBC, per 100 WBC | 0.96 | 2.19 | 0.99 | 0.66 | 0.241 | - |
| Platelets, ×10^3^ cells/uL | 830.5 | 760.5 | 776.1 | 46.54 | 0.471 | 192-832 |
| Mean platelet value, fl | 9.69 | 9.66 | 9.72 | 0.22 | 0.967 | 6.5-12.7 |
| WBC count, ×10^3^ cells/uL | 6.42 | 7.56 | 7.00 | 0.67 | 0.310 | 5.6-18.5 |
| Neutrophils, % of WBC | 23.96 | 30.15 | 33.01 | 2.98 | 0.102 | - |
| Lymphocytes, % of WBC | 70.12^a^ | 62.70^ab^ | 59.99^b^ | 2.86 | 0.046 | 26.2-82.9 |
| Monocytes, % of WBC | 6.11 | 6.86 | 7.17 | 1.61 | 0.625 | 1.4-8.3 |
| Eosinophils, % of WBC | 0.09 | 0.03 | 0.04 | 0.03 | 0.328 | 0-1.9 |
| Basophils, % of WBC | 0.42 | 0.50 | 0.27 | 0.11 | 0.326 | 0-0.90 |
| ^1^Abbreviations: CON, control; 3`-SL, 3`-sialyllactose; 6`-SL, 6`-sialyllactose; AST, aspartate aminotransferase; BUN, blood urea nitrogen; CPK, creatine phosphokinase; GGT, gamma-glutamyl transferase; GLDH, glutamate dehydrogenase; MCH, mean cell hemoglobin; MCHC, mean corpuscular hemoglobin; MCV, mean cell volume; RBC, red blood cell; SEM, standard error of the mean; WBC, white blood cell; SEM, standard error of the mean.  ^2^Estimated reference intervals for clinical chemistry and hematological outcomes for 30-d-old pigs (Ventrella et al., 2017).  ^3^Missing data-points reduced the sample size for various outcomes for reasons including missing samples or lack of data from the lab.  ^ab^Means lacking a common superscript letter within a row differ (*P* < 0.05). | | | | | | |

| **Supplemental Table 2.** Blood clinical chemistry and hematology outcomes at PND 61^1^ | | | | | | |
| --- | --- | --- | --- | --- | --- | --- |
|  | **Diet** | | | **Pooled SEM** |  | **Reference Interval^2^** |
| **Outcome** | **CON** | **3`-SL** | **6`-SL** |  | ***P*-value** |  |
| *n*^3^ | 11 | 12 | 10 | - | - | - |
| Clinical chemistry |  |  |  |  |  |  |
| Creatine, mg/mL | 0.76 | 0.79 | 0.76 | 0.03 | 0.412 | 0.51-1.39 |
| BUN, mg/dL | 6.57^b^ | 8.17^a^ | 7.41^ab^ | 0.38 | 0.004 | 4.0-39 |
| Total protein, g/dL | 4.81 | 4.57 | 4.66 | 0.16 | 0.474 | 2.5-6.6 |
| Albumin, g/dL | 3.28 | 3.11 | 3.19 | 0.09 | 0.304 | 1.9-4.0 |
| Globulin, g/dL | 1.53 | 1.46 | 1.36 | 0.12 | 0.602 | - |
| Albumin:globulin ratio | 2.23 | 2.23 | 2.31 | 0.17 | 0.917 | 0.7-2.2 |
| Calcium, mg/dL | 10.41 | 10.72 | 10.54 | 0.16 | 0.369 | - |
| Phosphorus, mg/dL | 9.04 | 8.60 | 8.50 | 0.27 | 0.232 | - |
| Sodium, mmol/L | 139.6 | 139.9 | 141.0 | 0.47 | 0.085 | 125-147 |
| Potassium, mmol/L | 5.13 | 5.08 | 4.98 | 0.19 | 0.677 | 2.9-4.6 |
| Sodium:potassium ratio | 27.17 | 27.67 | 28.57 | 1.03 | 0.315 | - |
| Chloride, mmol/L | 100.5 | 101.2 | 102.1 | 1.00 | 0.087 | - |
| Glucose, mg/dL | 120.6 | 120.7 | 122.3 | 6.68 | 0.899 | 34-159 |
| Total alkaline phosphatase, U/L | 293.7 | 261.9 | 193.7 | 37.40 | 0.097 | 110-1292 |
| AST, U/L | 26.57 | 48.00 | 53.26 | 13.04 | 0.270 | 13-65 |
| GGT, U/L | 61.53 | 54.83 | 50.67 | 8.89 | 0.606 | - |
| CPK, U/L | 923.7^b^ | 1,617^ab^ | 2,089^a^ | 437.4 | 0.047 | **-** |
| Cholesterol total mg/dL | 72.21 | 65.00 | 68.03 | 5.11 | 0.310 | - |
| GLDH, U/L | 0.59 | 1.00 | 1.18 | 0.21 | 0.109 | - |
| Bicarbonate (TCO_2_), mmol/L | 31.34 | 32.08 | 31.87 | 1.07 | 0.660 | - |
| Magnesium, mg/dL | 2.20 | 2.17 | 2.10 | 0.06 | 0.480 | - |
| Triglycerides, mg/dL | 28.19 | 23.25 | 24.66 | 3.57 | 0.357 | - |
| Anion gap | 12.82 | 11.92 | 12.24 | 0.90 | 0.358 | - |
| Hematology |  |  |  |  |  |  |
| RBC count, ×10^6^ cells/uL | 6.50 | 6.31 | 5.99 | 0.18 | 0.129 | 4.08-8.17 |
| Hemoglobin, g/dL | 10.05 | 9.79 | 9.39 | 0.25 | 0.090 | 4.32-13.3 |
| Hematocrit, % | 32.26 | 31.7 | 30.50 | 0.79 | 0.254 | 16-41 |
| MCV, fL | 49.74 | 50.25 | 51.55 | 0.85 | 0.067 | 34.2-61.3 |
| MCH, pg | 15.49 | 15.56 | 15.91 | 0.35 | 0.335 | 9.4-19.8 |
| MCHC, g/dL | 31.18 | 30.93 | 30.90 | 0.24 | 0.477 | 26.5-33.6 |
| Nucleated RBC, per 100 WBC | 1.50 | 1.50 | 1.00 | 0.31 | 0.255 | - |
| Platelets, ×10^3^ cells/uL | 621.0 | 590.3 | 555.9 | 36.30 | 0.426 | 192-832 |
| Mean platelet value, fl | 9.60 | 9.99 | 9.98 | 0.19 | 0.169 | 6.5-12.7 |
| WBC count, ×10^3^ cells/uL | 16.79 | 15.79 | 14.86 | 1.71 | 0.643 | 5.6-18.5 |
| Neutrophils % of WBC | 41.07 | 39.96 | 36.44 | 5.10 | 0.779 | - |
| Lymphocytes, % of WBC | 51.01 | 53.25 | 52.37 | 5.27 | 0.931 | 26.2-82.9 |
| Monocytes, % of WBC | 7.04^ab^ | 5.84^b^ | 9.61^a^ | 1.08 | 0.032 | 1.4-8.3 |
| Eosinophils, % of WBC | 0.10^b^ | 0.02^b^ | 0.28^a^ | 0.07 | 0.018 | 0-1.9 |
| Basophils, % of WBC | 0.36 | 0.55 | 0.66 | 0.21 | 0.350 | 0-0.90 |
| ^1^Abbreviations: CON, control; 3`-SL, 3`-sialyllactose; 6`-SL, 6`-sialyllactose; AST, aspartate aminotransferase; BUN, blood urea nitrogen; CPK, creatine phosphokinase; GGT, gamma-glutamyl transferase; GLDH, glutamate dehydrogenase; MCH, mean cell hemoglobin; MCHC, mean corpuscular hemoglobin; MCV, mean cell volume; RBC, red blood cell; SEM, standard error of the mean; WBC, white blood cell; SEM, standard error of the mean.  ^2^Estimated reference intervals for clinical chemistry and hematological outcomes for 30-d-old pigs (Ventrella et al., 2017).  ^3^Missing data-points reduced the sample size for various outcomes for reasons including missing samples or lack of data from the lab.  ^ab^Means lacking a common superscript letter within a row differ (*P* < 0.05). | | | | | | |

| **Supplemental Table 3.** Sialic acid concentrations in blood and brain samples at PND33^1,2^ | | | | | |
| --- | --- | --- | --- | --- | --- |
|  | **Diet** | | | **Pooled SEM** |  |
| **Outcome** | **CON** | **3`-SL** | **6`-SL** |  | ***P*-value** |
| *n* | 9 | 11 | 10 | - | - |
| Cerebellum |  |  |  |  |  |
| Neu5Ac, µg/g of wet tissue |  |  |  |  |  |
| Free | 57.49 | 50.92 | 58.84 | 7.68 | 0.411 |
| Bound | 920.7 | 921.7 | 958.6 | 54.41 | 0.537 |
| Total | 978.5 | 972.5 | 1,017 | 60.14 | 0.497 |
| Neu5Gc, µg/g of wet tissue |  |  |  |  |  |
| Free | 11.10 | 9.15 | 10.47 | 3.57 | 0.445 |
| Bound | 42.15 | 35.95 | 36.51 | 5.04 | 0.411 |
| Total | 52.87 | 47.00 | 47.00 | 3.03 | 0.286 |
| Hippocampus |  |  |  |  |  |
| Neu5Ac, µg/g of wet tissue |  |  |  |  |  |
| Free | 40.69 | 41.20 | 37.56 | 3.49 | 0.444 |
| Bound | 913.8 | 971.7 | 930.0 | 29.93 | 0.261 |
| Total | 959.0 | 1,015 | 970.5 | 32.86 | 0.268 |
| Neu5Gc, µg/g of wet tissue |  |  |  |  |  |
| Free | 4.81 | 5.71 | 6.22 | 0.61 | 0.289 |
| Bound | 34.12 | 33.82 | 31.88 | 3.11 | 0.840 |
| Total | 39.06 | 39.62 | 38.72 | 3.03 | 0.968 |
| Prefrontal cortex |  |  |  |  |  |
| Neu5Ac, µg/g of wet tissue |  |  |  |  |  |
| Free | 53.60 | 55.35 | 52.71 | 3.51 | 0.742 |
| Bound | 849.5 | 863.2 | 928.1 | 57.63 | 0.308 |
| Total | 906.0 | 918.5 | 980.3 | 55.30 | 0.337 |
| Neu5Gc, µg/g of wet tissue |  |  |  |  |  |
| Free | 6.46 | 6.48 | 6.58 | 0.69 | 0.990 |
| Bound | 31.63 | 31.15 | 30.25 | 3.96 | 0.921 |
| Total | 38.02 | 37.28 | 40.23 | 3.11 | 0.634 |
| Striatum |  |  |  |  |  |
| Neu5Ac, µg/g of wet tissue |  |  |  |  |  |
| Free | 61.09 | 55.95 | 55.54 | 6.66 | 0.347 |
| Bound | 765.5 | 777.2 | 746.1 | 44.89 | 0.631 |
| Total | 826.0 | 833.2 | 803.0 | 40.71 | 0.653 |
| Neu5Gc, µg/g of wet tissue |  |  |  |  |  |
| Free | 4.92 | 4.61 | 4.13 | 0.52 | 0.544 |
| Bound | 43.66 | 39.98 | 41.45 | 3.64 | 0.538 |
| Total | 47.01 | 45.49 | 42.99 | 3.90 | 0.627 |
| Plasma^3^ |  |  |  |  |  |
| Neu5Ac, µg/ml |  |  |  |  |  |
| Free | 4.50 | 3.93 | 4.52 | 1.43 | 0.940 |
| Bound | 557.7 | 564.8 | 527.3 | 26.83 | 0.355 |
| Total | 561.5 | 567.6 | 532.1 | 27.29 | 0.392 |
| Neu5Gc, µg/ml |  |  |  |  |  |
| Free | 1.75 | 1.70 | 1.60 | 0.20 | 0.802 |
| Bound | 96.34 | 95.79 | 91.20 | 5.21 | 0.390 |
| Total | 97.97 | 97.44 | 92.99 | 5.33 | 0.415 |
| ^1^Abbreviations: Neu5Ac, *N*-acetylneuraminic acid; Neu5Gc, *N*-glycolylneuraminic acid; SEM, standard error of the mean; PND, postnatal day; CON, control; 3`-SL, 3`-sialyllactose; 6`-SL, 6`-sialyllactose.  ^2^Bound sialic acid concentrations calculated as the difference between total and free concentrations.  ^3^One pig was excluded due to a missing blood sample resulting in 9 pigs in the 6`-SL group. | | | | | |

| **Supplemental Table 4.** Sialic acid concentrations in blood and brain samples at PND 61^1,2^ | | | | | |
| --- | --- | --- | --- | --- | --- |
|  | **Diet** | | | **Pooled SEM** |  |
| **Outcome** | **CON** | **3`-SL** | **6`-SL** |  | ***P*-value** |
| *n* | 11 | 12 | 10 | - | - |
| Cerebellum |  |  |  |  |  |
| Neu5Ac, µg/g of wet tissue |  |  |  |  |  |
| Free | 50.14 | 54.72 | 49.22 | 3.32 | 0.357 |
| Bound | 891.8 | 909.0 | 902.2 | 67.92 | 0.896 |
| Total | 939.7 | 964.7 | 951.0 | 67.71 | 0.797 |
| Neu5Gc, µg/g of wet tissue |  |  |  |  |  |
| Free | 5.73 | 7.50 | 5.65 | 1.02 | 0.277 |
| Bound | 31.54 | 31.50 | 35.33 | 3.40 | 0.452 |
| Total | 38.07 | 39.55 | 39.90 | 2.46 | 0.614 |
| Hippocampus |  |  |  |  |  |
| Neu5Ac, µg/g of wet tissue |  |  |  |  |  |
| Free | 37.77 | 39.85 | 38.87 | 2.44 | 0.714 |
| Bound | 943.7 | 970.9 | 960.4 | 37.86 | 0.787 |
| Total | 981.5 | 1,011 | 999.3 | 39.11 | 0.769 |
| Neu5Gc, µg/g of wet tissue |  |  |  |  |  |
| Free | 4.66 | 5.71 | 5.74 | 0.44 | 0.152 |
| Bound | 38.17 | 35.12 | 33.55 | 1.59 | 0.129 |
| Total | 42.46 | 40.81 | 39.74 | 1.76 | 0.439 |
| Prefrontal Cortex |  |  |  |  |  |
| Neu5Ac, µg/g of wet tissue |  |  |  |  |  |
| Free | 57.92 | 52.94 | 52.77 | 3.71 | 0.363 |
| Bound | 864.7 | 861.9 | 881.3 | 46.40 | 0.927 |
| Total | 922.9 | 914.8 | 933.9 | 44.96 | 0.938 |
| Neu5Gc, µg/g of wet tissue |  |  |  |  |  |
| Free | 5.37 | 5.27 | 4.96 | 0.72 | 0.866 |
| Bound | 31.69 | 31.53 | 33.56 | 1.81 | 0.668 |
| 403Total | 37.02 | 36.94 | 37.38 | 1.75 | 0.981 |
| Striatum |  |  |  |  |  |
| Neu5Ac, µg/g of wet tissue |  |  |  |  |  |
| Free | 55.39 | 53.27 | 54.56 | 3.12 | 0.870 |
| Bound | 638.0 | 684.1 | 630.8 | 27.65 | 0.121 |
| Total | 696.0 | 738.6 | 686.3 | 27.97 | 0.147 |
| Neu5Gc, µg/g of wet tissue |  |  |  |  |  |
| Free | 3.57 | 4.45 | 3.73 | 0.45 | 0.351 |
| Bound | 42.93 | 48.02 | 43.09 | 3.71 | 0.278 |
| Total | 48.19 | 52.34 | 49.42 | 3.72 | 0.278 |
| Plasma |  |  |  |  |  |
| Neu5Ac, µg/ml |  |  |  |  |  |
| Free | 5.94 | 6.56 | 6.83 | 0.36 | 0.095 |
| Bound | 503.0^a^ | 412.2^b^ | 427.9^b^ | 30.51 | 0.022 |
| Total | 509.1^a^ | 418.6^b^ | 434.4^b^ | 30.45 | 0.022 |
| Neu5Gc, µg/ml |  |  |  |  |  |
| Free | 1.67 | 2.04 | 1.96 | 0.17 | 0.055 |
| Bound | 102.7 | 102.2 | 105.9 | 5.28 | 0.823 |
| Total | 104.5 | 104.2 | 107.9 | 5.28 | 0.822 |
| ^1^Abbreviations: Neu5Ac, *N*-acetylneuraminic acid; Neu5Gc, *N*-glycolylneuraminic acid; SEM, standard error of the mean; PND, postnatal day; CON, control; 3`-SL, 3`-sialyllactose; 6`-SL, 6`-sialyllactose.  ^2^Bound sialic acid concentrations calculated as the difference between total and free concentrations.  ^ab^Means lacking a common superscript letter within a row differ (*P* < 0.05). | | | | | |
